# A Cross-Sectional Study of Disclosure of HIV Status to Children and Adolescents in Western Kenya

**DOI:** 10.1371/journal.pone.0086616

**Published:** 2014-01-27

**Authors:** Rachel C. Vreeman, Michael L. Scanlon, Ann Mwangi, Matthew Turissini, Samuel O. Ayaya, Constance Tenge, Winstone M. Nyandiko

**Affiliations:** 1 Children’s Health Services Research, Department of Pediatrics, Indiana University School of Medicine, Indianapolis, Indiana, United States of America; 2 USAID-Academic Model Providing Access to Healthcare (AMPATH), Eldoret, Kenya; 3 Department of Behavioral Science, School of Medicine, College of Health Sciences, Moi University, Eldoret, Kenya; 4 Department of Child Health and Paediatrics, School of Medicine, College of Health Sciences, Moi University, Eldoret, Kenya; Alberta Provincial Laboratory for Public Health/University of Alberta, Canada

## Abstract

**Introduction:**

Disclosure of HIV status to children is essential for disease management but is not well characterized in resource-limited settings. This study aimed to describe the prevalence of disclosure and associated factors among a cohort of HIV-infected children and adolescents in Kenya.

**Methods:**

We conducted a cross-sectional study, randomly sampling HIV-infected children ages 6–14 years attending 4 HIV clinics in western Kenya. Data were collected from questionnaires administered by clinicians to children and their caregivers, supplemented with chart review. Descriptive statistics and disclosure prevalence were calculated. Univariate analyses and multivariate logistic regression were performed to assess the association between disclosure and key child-level demographic, clinical and psychosocial characteristics.

**Results:**

Among 792 caregiver-child dyads, mean age of the children was 9.7 years (SD = 2.6) and 51% were female. Prevalence of disclosure was 26% and varied significantly by age; while 62% of 14-year-olds knew their status, only 42% of 11-year-olds and 21% of 8-year-olds knew. In multivariate regression, older age (OR 1.49, 95%CI 1.35–1.63), taking antiretroviral drugs (OR 2.27, 95%CI 1.29–3.97), and caregiver-reported depression symptoms (OR 2.63, 95%CI 1.12–6.20) were significantly associated with knowing one’s status. Treatment site was associated with disclosure for children attending one of the rural clinics compared to the urban clinic (OR 3.44, 95%CI 1.75–6.76).

**Conclusions:**

Few HIV-infected children in Kenya know their HIV status. The likelihood of disclosure is associated with clinical and psychosocial factors. More data are needed on the process of disclosure and its impact on children.

## Introduction

In 2011, the World Health Organization (WHO) estimated there were 3.4 million children under 15 years of age living with the Human Immunodeficiency Virus (HIV), while an estimated 330,000 children were newly infected in 2011 alone. [1] The advent of antiretroviral therapy (ART) and expanded access to treatment have resulted in more HIV-infected children reaching adolescence and adulthood, [2] especially in resource-limited settings like sub-Saharan Africa, which is home to over 90% of the pediatric HIV-infected population. [1] As HIV-infected children live longer, emerging challenges to comprehensive pediatric HIV care include supporting high rates of adherence to treatment, preventing secondary transmission and promoting overall physical and mental health. [3] For these children, learning about their HIV diagnosis - often referred to as disclosure - is an important step towards long-term disease management and necessary for the transition from pediatric care into adolescent and adult care settings. [4].

In the United States, recommendations for disclosure of HIV status to children endorse a gradual process of giving age-appropriate information as the child develops the cognitive and emotional maturity to process this information. [5] Globally, institutions such as the WHO have issued similar guidelines, [6] but there are few published data on standardized, culturally appropriate disclosure protocols in resource-limited settings. A recent review on disclosure of HIV status to children found that lower proportions of children in low- and middle-income countries (LMIC) knew their status compared to those in high-income countries and among those that did know, children in LMIC reported learning it at older ages. [7] Of the 21 studies included for review by Pinzon-Iregui et al. that reported prevalence of disclosure, median prevalence of disclosure among similarly aged children was 20% in studies conducted in LMIC and 43% in high-income countries, while median age of disclosure was 9.6 years in LMIC and 8.3 years in high-income countries. Caregivers in both resource-poor and resource-rich settings report weighing the potential risks and benefits of disclosure. While the child’s increasing age, independence and concerns about medication adherence may motivate caregivers to disclose, caregivers often have fears about the negative emotional effects of disclosure and HIV-related stigma and discrimination. Few studies have measured the actual impact of disclosure on children’s clinical, emotional and psychosocial outcomes. [8] Anecdotal evidence from qualitative and quantitative studies suggests both positive and negative effects of disclosure on disease progression, adherence to ART, caregiver-child relationships, access to social support and psychological health outcomes.

As children in HIV care systems mature through adolescence, more data on disclosure of their HIV status in resource-limited settings are needed. These data will help inform the design and adoption of culturally-relevant guidelines which providers and other health professionals can use to support caregivers and children through this difficult process. Previously, we reported the results of a pilot study on the prevalence of disclosure among 270 HIV-infected children at a single urban clinic in western Kenya [9] under the umbrella of the Academic Model Providing Access to Healthcare (AMPATH), one of the largest HIV care systems in sub-Saharan Africa. This article describes the results of the parent study that assessed the prevalence of disclosure and factors associated with disclosure among a larger sample drawn from 4 urban and rural AMPATH clinics across western Kenya.

## Methods

### Study Design

**Figure 1:**
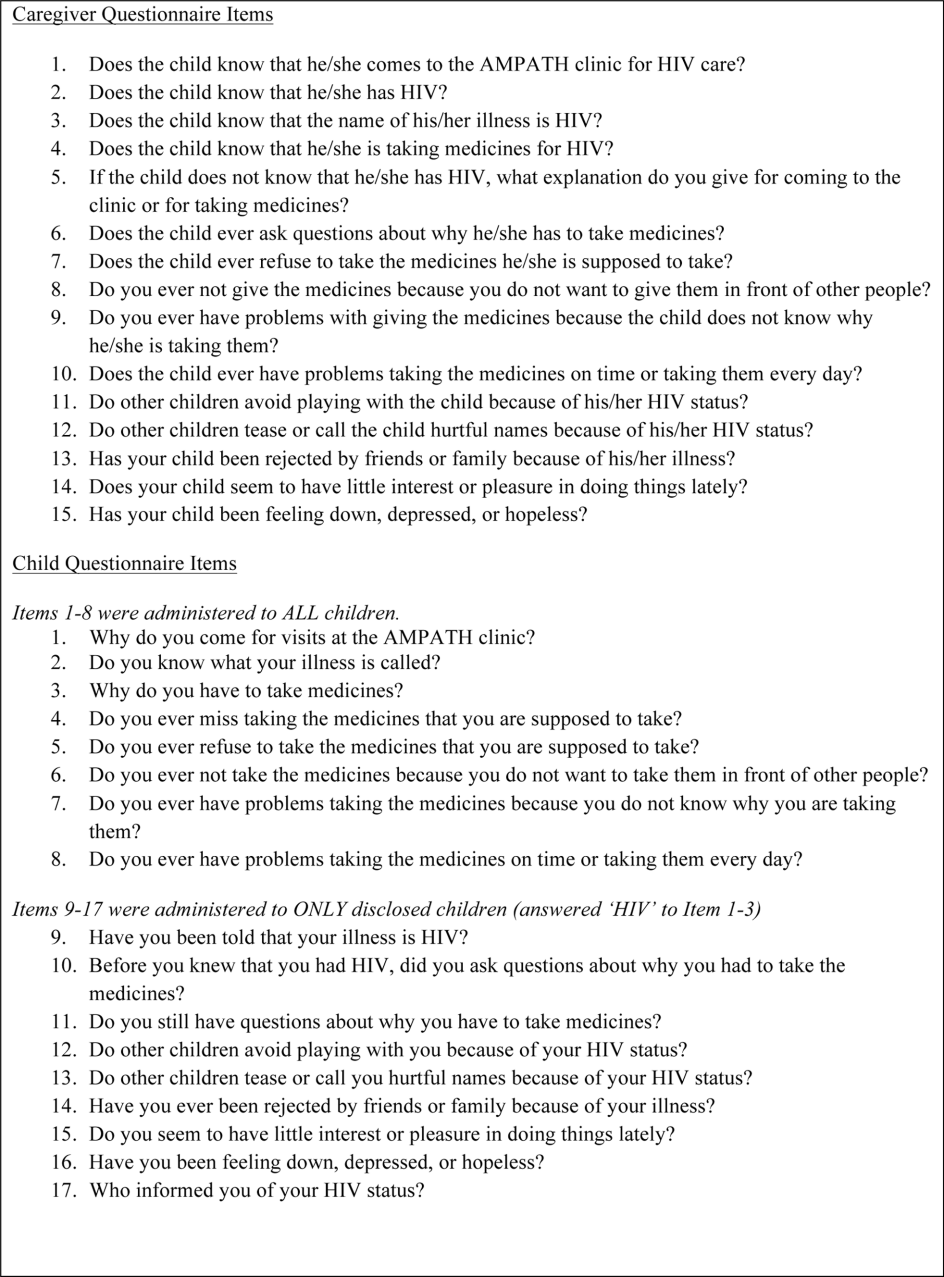
Disclosure questionnaire items.

We conducted a cross-sectional study using assessments of a random sample of caregivers and their HIV-infected children ages 6–14 years receiving care at four AMPATH clinics in western Kenya. Clinicians independently administered a 17-item questionnaire to HIV-infected children and a 15-item questionnaire to their caregivers at routine clinic visits to assess disclosure status, ART adherence, stigma and depression (see Figure 1 for full set of caregiver and child questionnaire items on the Disclosure Questionnaire). Children were asked to leave the examination room when clinicians were administering the questionnaire to caregivers to avoid accidental disclosure. All children were asked general questions about reasons for receiving care and disclosure status, but only children who self-reported knowing their HIV status were asked questions about HIV-related stigma and depression. Caregivers were asked to respond to HIV-related stigma and depression as experienced by their children. Depression symptoms were evaluated using the PHQ-2 questions, [10] but other questionnaire items were developed in this setting. Demographic and clinical characteristics of child participants were extracted from chart review. Age, weight, orphan status, medications, and duration of enrollment in AMPATH were calculated at study visit. The most recent CD4 count and CD4 percentage (CD4%) in a child’s medical chart was used. No demographic or other data were collected for caregivers. The study was approved by the Institutional Review Board at Indiana University School of Medicine in Indianapolis, Indiana, USA and by the Institutional Research and Ethics Committee at Moi University School of Medicine in Eldoret, Kenya. Consent and assent were waived for this study as the questionnaires were administered during routine visits with clinicians and the assessments were in line with AMPATH’s protocol to begin routine collection of disclosure data. Both the Institutional Review Board at Indiana University School of Medicine and the Institutional Research and Ethics Committee at Moi University School of Medicine approved the waiving of consent and assent for this study. Data were collected from July 2011 to June 2012.

### Setting

AMPATH is a partnership between Moi University School of Medicine, Moi Teaching and Referral Hospital (MTRH) and a consortium of North American academic medical centers led by Indiana University. [11] As of January 2013, AMPATH provides comprehensive HIV care, including free ART and psychosocial and nutritional support, to over 55,000 HIV-infected adults and 15,000 pediatric patients in 56 clinics and satellite sites across western Kenya. AMPATH’s protocol on disclosure of HIV status to children recommends initiating disclosure for all children who are 10 years and above but the decision to disclose is ultimately left to the child’s caregiver. Caregivers are invited to private and group disclosure counseling sessions and offered support at the clinic that includes information about HIV, discussing worries, fears and potential advantages of disclosure and making a disclosure plan. Children over 14 years of age, those without caregivers and those at risk of endangering themselves through poor adherence or others through sexual activity are often identified for enhanced disclosure counseling.

### Study Participants

The study population was caregivers and their HIV-infected children ages 6 to 14 years who were enrolled in care at 4 AMPATH clinics: MTRH, Kitale, Turbo, and, Webuye. These clinics were selected because they are among AMPATH’s largest pediatric sites and treat geographically and ethnically diverse patient populations. A “caregiver” was defined as someone responsible for the well-being of the child, who brought the child to clinic, and who was knowledgeable about the child’s HIV care behaviors (e.g., adherence to ART). HIV infection was defined as having one positive HIV DNA PCR test or one positive HIV ELISA antibody test. A patient randomization module within the electronic health record system was used to select a random sample of HIV-infected patients ages 6 to 14 years enrolled in care at the 4 study clinics. Disclosure status was not recorded in the electronic data and was not considered in the inclusion criteria. The minimum age limit was based on a previous pilot study that included a subset of this population [9] while the maximum age limit was selected because children aged 15 years and above are often treated in adult care settings where HIV disclosure is assumed. No incentives were provided to study participants for participation.

### Outcomes

The outcome variable was children’s disclosure status, defined as a binomial variable of “disclosed” versus “not disclosed.” Children were considered disclosed if the caregiver answered “yes” to any of the questions about the child knowing about their HIV status (see Figure 1, Caregiver Items 1–4) or if the child reported HIV as the reason he/she comes to clinic or takes medications, the name of his/her illness or if he/she reported being told their illness is HIV (see Figure 1, Child Items 1–3, 9).

### Data Analysis

Descriptive statistics were calculated and the prevalence of disclosure was described for child participants and within subcategories by age. Univariate analyses with Pearson’s chi-squared (χ^2^) tests were used to investigate associations between a child’s disclosure status and child-level demographic, clinical and psychosocial characteristics. Multivariate analyses were then conducted using logistic regression with odds ratios (OR) and 95% confidence intervals (95%CI). As this was a largely exploratory analysis, we included the entire set of variables in the multivariate model whether they were significant in univariate analyses or not. Binomial variables were calculated to describe child-level adherence, stigma and depression based on child and caregiver responses.

“Non-adherence” was defined as any missed doses in the past 30 days by caregiver-report or child-report on the standard AMPATH clinical encounter form or any indication of adherence difficulties reported by caregivers (see Figure 1, Caregiver Items 7–10) or children (see Figure 1, Child Items 4–8) on the Disclosure Questionnaire. “Stigma” was defined as any indication of child-experienced stigma from the caregiver (see Figure 1, Caregiver Items 11–13) or the child (see Figure 1, Child Items 12–14) and “depression” was defined by any indication of child depression symptoms as reported by the caregiver (see Figure 1, Caregiver Items 14, 15) or the child (see Figure 1, Child Items 15, 16) using the PHQ-2. All statistical analyses were performed using STATA version 10.0 (StataCorp LP, College Station, Texas, USA).

## Results

### Characteristics of Child Participants

Among 792 children, mean age was 9.8 years (SD = 2.6) and 51% were female (Table 1). Children had a mean weight-for-age Z-score (WAZ) of −1.3 (SD = 1.2). Almost half of the children were orphans (48%) with orphan defined as having a deceased biological mother, having a deceased biological father or having both. The biological mother was the caregiver for a little over half of the children (60%). Most children were on ART (79%), while only 16 children (2%) were also taking anti-tuberculosis medication. Children had a mean duration of enrollment in an AMPATH clinic of 48 months (SD = 25.3) and mean CD4% of 28% (SD = 0.16). Only 8% of children had indications of non-adherence to ART on the standard clinical encounter form.

**Table 1:** Demographic and Clinical Characteristics of Child Participants by Disclosure Status.

	Disclosed No (N = 588)		Disclosed Yes (N = 204)		P-Value†
Variable	N	%	N	%	
**Gender**					
Female	298	51%	107	52%	0.663
Male	290	49%	97	48%	
**On ART**					
Yes	450	24%	174	86%	0.006*
No	138	76%	29	14%	
**On Anti-TB**					
Yes	14	2%	2	1%	0.221
No	572	98%	201	99%	0.221
**WHO Stage**					
1	135	23%	64	32%	0.079
2	142	24%	41	20%	
3	271	46%	89	44%	
4	39	7%	9	4%	
**Orphan Status**					
Both parents living	325	55%	91	45%	0.043*
Both parents dead	70	12%	35	17%	
Mother dead	57	10%	23	11%	
Father dead	79	13%	28	19%	
Do not know	57	10%	17	8%	
**Caregiver**					
Mother	347	59%	104	51%	0.382
Father	56	9%	19	9%	
Aunt/Uncle	69	125	28	14%	
Grandparent	52	9%	19	9%	
Sibling	18	3%	9	5%	
Children’s Home	15	3%	8	4%	
Other	31	5%	17	8%	
**Ethnic Group**					
Kalenjin	168	29%	36	17%	0.001*
Kikuyu	69	12%	18	9%	
Luhya	265	45%	126	62%	
Luo	47	8%	12	6%	
Other	39	6%	12	6%	
**Clinic**					
MTRH	223	38%	47	23%	<0.001*
Kitale	123	21%	44	22%	
Turbo	132	22%	40	19%	
Webuye	110	19%	73	36%	
**Adherence on Clinic Encounter** **(30-day recall)**					
Adherent	542	92%	185	91%	0.504
Non-adherent	46	8%	19	9%	
	**Mean**	**SD**	**Mean**	**SD**	
**Age (years)**	9.4	2.2	11.4	2.3	<0.001*
**Time enrolled at AMPATH clinic (months)**	47.9	24.9	47.6	24.9	0.967
**CD4 Count**	793.5	453.4	712.3	386.3	0.035*
**CD4%**	0.29	0.18	0.27	0.10	0.582

† Univariate analyses using Pearson’s chi-squared tests.

* Significant at the p<0.05 level.

### Prevalence of Disclosure

**Figure 2:**
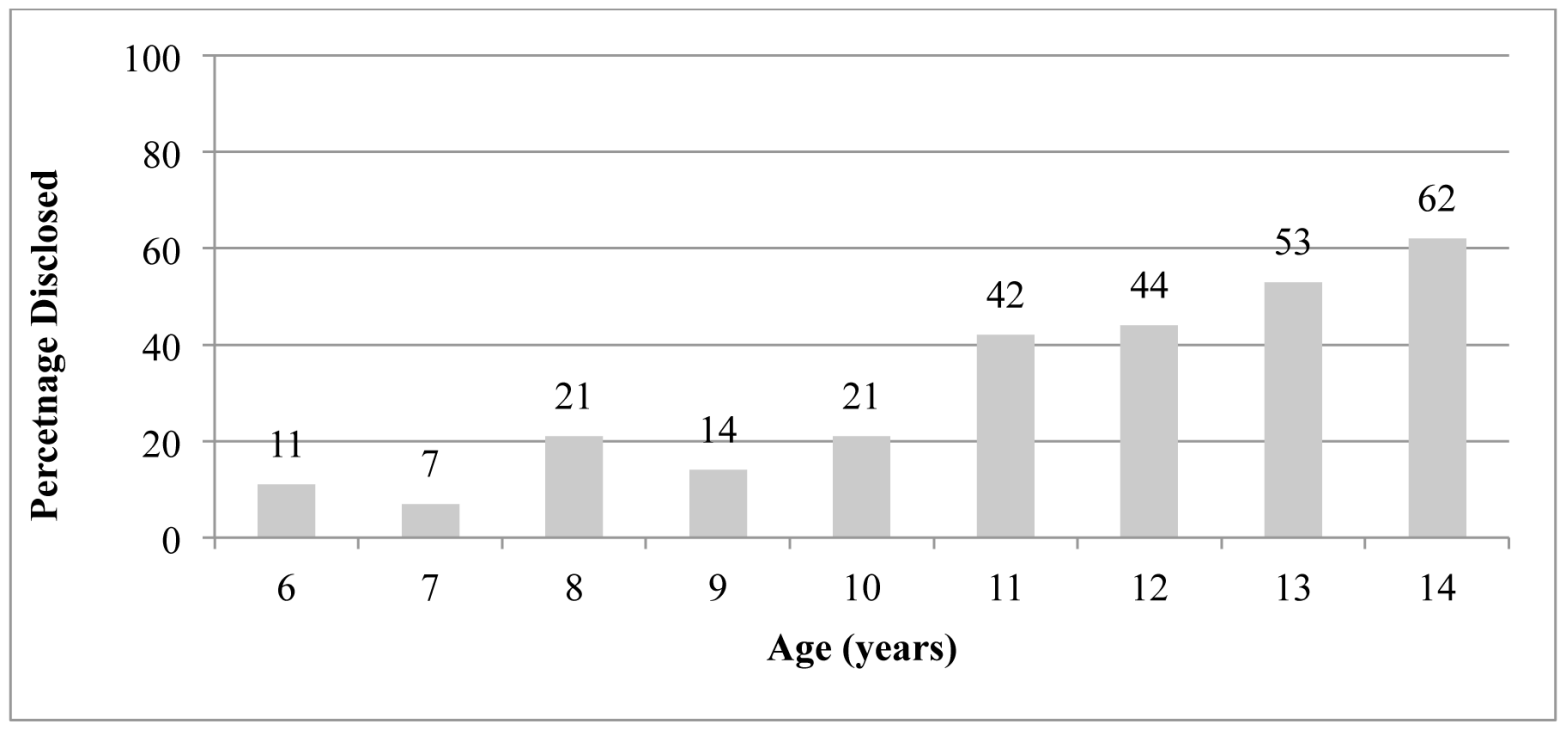
Prevalence of disclosure by age.

The overall prevalence of disclosure was 26%. The proportion of children who knew their status was greater among older children compared to young children. Disclosure by age is shown in Table 1. While only 9% of 6- to 7-year olds knew their status, 33% of 10- to 11-year olds and 56% of 13- to 14-year olds reported knowing their status (Figure 2). The prevalence of disclosure also differed by clinic: disclosure prevalence was highest at Webuye (40%) and lowest at MTRH (17%).

### Association between Disclosure and Child Characteristics

In univariate analyses, older age (p<.01), being an orphan (p = .04), having a lower CD4 count (p = .03), being on ART (p = .01), ethnic group (p<.01) and treatment site (p<.01) were all significantly associated with knowing one’s status (Table 1). While disclosure status was not associated with adherence either reported on the clinical encounter form or by caregivers, disclosure was associated with child-reported adherence (p = .03) with disclosed children reporting more non-adherence than non-disclosed children (Table 2). Caregiver-reported child-experienced stigma and child depression symptoms were both significantly associated with disclosure; while only 2% caregivers of non-disclosed children reported stigma and 4% reported depression symptoms, 10% of caregivers of disclosed children reported stigma (p<.01) and 12% reported depression symptoms (p<.01).

**Table 2:** Indicators of Adherence, Stigma and Depression by Caregiver- and Child-Report.

	Disclosed No (N = 588)		Disclosed Yes (N = 204)		P-Value†
Variable	N	%	N	%	
**Caregiver-Reported Variables:**					
**Combined Adherence**					
Adherent	280	48%	92	45%	0.534
Non-adherent	308	52%	112	55%	
**Combined Stigma**					
No reported stigma	575	98%	184	90%	<0.001*
Reported stigma	13	2%	20	10%	
**Combined Depression**					
No reported depression	566	96%	180	88%	<0.001*
Reported depression	22	4%	24	12%	
**Child-Reported Variables:**					
**Combined Adherence**					
Adherent	488	83%	155	76%	0.027*
Non-adherent	100	17%	49	24%	
**Combined Stigma** **					
No reported stigma	–	–	189	93%	–
Reported stigma	–	–	15	7%	–
**Combined Depression** **	–	–			–
No reported depression	–	–	195	96%	–
Reported depression	–	–	9	4%	–

† Univariate analyses using Pearson’s chi-squared tests.

* Significant at the p<0.05 level.

** Only disclosed children were asked questions about stigma and depression.

In multivariate analyses, variables significantly associated with disclosure were a child’s older age (OR 1.5, 95%CI 1.3–1.6), being on ART (OR 2.3, 95%CI 1.3–4.0), and caregiver-reported child depression symptoms (OR 2.6, 95%CI 1.1–6.2) (Table 3). Treatment site was also associated with disclosure at two clinics; being treated at Webuye compared to MTRH was significantly associated with disclosure (OR 3.4, 95%CI 1.7–6.8). Children with a deceased father tended to be more likely to know their status than non-orphans (OR 1.6, 95%CI 0.9–2.8), as did children with caregivers who reported experiences of HIV stigma (OR 2.4, 95%CI 0.9–6.2), but neither test reached statistical significance. Gender, primary caregiver, CD4%, duration enrolled in AMPATH, malnutrition and adherence were not associated with disclosure in multivariate regression.

**Table 3:** Factors Associated with Disclosure Status in Multivariate Regression Model.

Variable	Odds Ratio	95% Confidence Interval
Female vs. Male	0.81	0.55–1.20
Age	1.49	1.35–1.63*
On ART (Yes vs. No)	2.26	1.29–3.97*
On Anti-TBs (Yes vs. No)	0.15	0.01–2.50
Time enrolled at AMPATH clinic	1.00	0.99–1.01
CD4%	0.54	0.14–2.05
**Orphan Status**		
Total orphan vs. Non-orphan	1.19	0.49–2.90
Mother dead vs. Non-orphan	0.87	0.39–1.97
Father dead vs. Non-orphan	1.62	0.92–2.85
Parent status unknown vs. Non-orphan	1.35	0.53–3.48
**Malnutrition**		
Mild malnutrition vs. Normal	1.06	0.66–1.71
Moderate malnutrition vs. Normal	1.07	0.61–1.89
Severe malnutrition vs. Normal	0.75	0.26–2.16
**Disease Stage**		
WHO Stage II vs. WHO Stage I	0.62	0.35–1.09
WHO Stage III vs. WHO Stage I	0.72	0.43–1.21
WHO Stage IV vs. WHO Stage I	0.41	0.15–1.08
**Ethnic Group**		
Kikuyu vs. Kalenjin	1.42	0.66–3.12
Luhya vs. Kalenjin	1.66	0.95–2.90
Luo vs. Kalenjin	1.74	0.72–4.20
**Clinic**		
Webuye vs. MTRH	3.44	1.75–6.76*
Kitale vs. MTRH	1.94	0.96–3.92
Turbo vs. MTRH	1.50	0.76–2.95
**Caregiver-reported variables**		
Non-adherent vs. Adherent	1.31	0.86–1.98
Reported stigma vs. No reported stigma	2.39	0.93–6.18
Reported depression vs. No reported depression	2.63	1.12–6.20*

* Significant in multivariate regression (95%CI does not include 1.00).

### Child versus Caregiver-reported Variables

Child-reported versus caregiver-reported variables related to disclosure status, adherence to ART, and experiences of stigma and depression were analyzed to identify discrepancies. Caregivers were more likely to report that the child knew their HIV status (p<.01), had poor adherence (p<.01), and had experiences with HIV-related stigma (p<.01) and depression symptoms (p<.01) compared to children’s self-reports.

## Discussion

As children with HIV survive into adolescence and adulthood at unprecedented rates, disclosure of HIV status is an essential component of pediatric HIV care and long-term disease management. This study investigated the prevalence and correlates of disclosure of HIV status to children in 4 clinics in western Kenya. We found a minority of children aged 6–14 years knew their status, consistent with findings from studies in Ghana, [12] Uganda, [13] and a previous study in Nairobi, Kenya, which found prevalence of disclosure to be 19% among 271 children with a median age of 9 years. [14] We did, however, find higher rates of disclosure in this expanded sample compared to rates of disclosure in a pilot study that revealed only 11% of children (median age 9.3 years) knew their HIV status. The results of this study prompted a program-wide reevaluation of AMPATH disclosure protocols and retraining of clinic-level staff. We are also now in the process of evaluating a 2–year disclosure intervention that includes further training of disclosure staff, employment of dedicated disclosure counselors and tailored disclosure curricula and materials.

Our study revealed a number of associations between disclosure status and demographic and clinical characteristics. Older children knew their status more frequently than younger children, likely as a result of increasing maturity, independence and responsibility for self-care that required knowledge of their status. For example, our finding that those on ART were significantly more likely to know their status may reflect disclosure following increased disease management activities like taking ART. We did not find any associations between disclosure status and clinical indicators like CD4 count and WHO disease stage. A study among Thai adolescents found that while disclosure was associated with CD4% below 30% in multivariate analysis, disclosure status was not associated with virologic outcomes. [15] In contrast, a study in Romania found that children who did not know their HIV status were at higher risk for disease progression, measured by CD4 count decline and death compared to disclosed children. [16] Other clinic-level factors like retention in care may also be associated with disclosure status and are important to understand, however, we did not evaluate retention in care in this study.

The relationship between adherence to ART and disclosure is not well described and studies report mixed results.[17]–[19] There are several reasons disclosure might be associated with non-adherence. Disclosure is a traumatic event for many children and can be accompanied by feelings of anger, hopelessness and rebellion, which may lead to temporary or longer-term adherence problems. The negative effects of HIV-related stigma, including efforts to keep the diagnosis secret by hiding or not taking medicines, may also impact adherence to therapy for disclosed children more than non-disclosed children. Adherence issues may be compounded by other adolescent-specific factors such as increased incidence of depression [20] and generally poorer medication adherence among this age group. [21] On the other hand, there are also reasons to believe disclosure may lead to improved adherence, including increased responsibility over medication-taking and better access to social support. Pediatric HIV providers often recommend disclosure of HIV status to children as necessary to building trusting provider-patient and family relationships and developing disease management skills that facilitate adherence. [22] In the only longitudinal study to assess adherence pre- and post-disclosure, Blasini et al reported that approximately 58% of children and their caregivers reported that adherence improved post-disclosure; however, adherence was assessed by self- and proxy-report among a small sample of only 40 children and clinicians felt that adherence improved in only 25% of cases. [23] Furthermore, since the study assessed disclosure after an intensive, supportive disclosure intervention, its results may not be representative of the majority of disclosure experiences.

Our finding that reports of adherence differed significantly depending on whether adherence was caregiver-reported or child-reported is indicative of the ongoing difficulties of clinic-level staff in resource-limited settings to accurately assess adherence to ART. A systematic review on adherence to ART found that adherence assessment items are rarely validated, that proxy-reports (i.e., caregiver-reports) often overestimate adherence and that children report more non-adherence than their caregivers do. [24] In our study, children reported less non-adherence than their caregivers, but these findings may be shaped by several cultural-specific biases. In particular, children in this setting with strong cultural traditions requiring children to obey authority figures (i.e., caregivers and clinicians) may be more vulnerable to social desirability pressure to report higher adherence. [25] In addition, despite clinical protocols recommending private interviewing of children about adherence, children are seldom questioned in private as was required for completion of the evaluations within this study. Finally, many of the children involved in this study were in the care of their grandparents and other extended family members rather than their biological parents. These non-parent caregivers may feel less pressure to report adherence.

Few studies investigate the psychosocial impact of disclosure in resource-limited settings. While this study was not designed to assess the impact by pre- and post-disclosure characteristics, we found higher rates of experiences of HIV-related stigma and depression symptoms among disclosed children, although only depression symptoms were significantly associated with disclosure in multivariate regression. This finding contradicts the findings of studies from the US and Zambia that suggest non-disclosed children have increased levels of psychological distress, including anxiety and depression, internalizing behavioral problems and poorer psychological adjustment compared to children that know their status.[26]–[28] In one of the few longitudinal studies measuring the psychosocial impact of disclosure, Butler et al found no significant association between caregiver-reported quality of life indicators pre- and post-disclosure among 395 perinatally HIV-infected children in the US Pediatric AIDS Clinical Trials Group 219C. [29] Our findings highlight the need to investigate the impact of disclosure on emotional and mental health outcomes in settings like Kenya so that appropriate support services can be provided.

Significant variations in the prevalence of disclosure among the clinics included in this study deserve further attention. Our analyses may not capture significant clinic-level factors, such as clinic staff motivated or experienced in disclosure; cultural factors such as varying populations of ethnic groups with differing perspectives on disclosure; or other structural factors like urban versus rural characteristics and transportation time and cost to clinic. In our sample, the prevalence of disclosure was highest at Webuye (39.9%), one of the rural satellite clinics, and was significantly lower at MTRH (17.4%), the second largest referral hospital in the country located in an urban center. Interestingly, caregivers of children attending Webuye clinic also reported significantly higher medication adherence (63.4%) than caregivers of children at MTRH (28.8%). We are aware of at least one nurse counselor at the Webuye clinic who expressed high interest in disclosure and its impact on care, which may contribute to the clinic’s higher rates of disclosure. While AMPATH clinics routinely offer counseling (including disclosure counseling) and support group services to children and their caregivers, we did not investigate differences in clinic-level services or their utilization by study group participants. Identifying clinic-level factors that promote or impede disclosure may help shape best practices for pediatric HIV care.

Many factors influence how and when caregivers decide to disclose to a child. This study did not assess caregiver perspectives on disclosure; however, previous qualitative work in this setting found that caregivers of HIV-infected children weighed potential risks and benefits as they made their decisions about when to disclose. [30] Perceived risks in this setting included the child being too young to understand, negative emotional consequences for the child and the subsequent disclosure of the child’s status to others, resulting in stigma and discrimination. At least one study found that children who disclosed their status to friends over the study period showed greater improvements in CD4 cell counts than children who had not disclosed, which may suggest better health outcomes after engaging social support. [31] On the other hand, caregivers believed that disclosure might lead to positive changes, including the child asking fewer questions, improved adherence to medications, and better access to social support. These findings are consistent with perspectives of caregivers in other resource-rich and resource-limited settings, who identify similar risks and benefits of disclosure. [7] While not significant, we found some indication that disclosure status varies by ethnic group in our setting in western Kenya. More qualitative data are needed to further explore how cultural beliefs may impact decisions about how and when to disclose HIV status to children in this setting.

This study had a number of limitations for consideration. One limitation was that we did not assess the prevalence of partial disclosure, where a child has incomplete information about HIV, which may be part of an age-appropriate disclosure process. [32] We counted any knowledge of HIV as a reason for treatment as disclosure. In other cases, caregivers or healthcare providers may give inaccurate information regarding the child’s diagnosis, such as attributing the illness, medication or clinic responsibilities to a different, often less-stigmatized condition like tuberculosis. Investigating how partial disclosure and misinformation are used by caregivers and healthcare providers and the impact on full disclosure, clinical outcomes, and psychosocial outcomes are important to understand. We also did not consider whether time since diagnosis or duration on ART were associated with disclosure of HIV status. In this setting, the vast majority of children are perinatally infected and thus diagnosed at birth or shortly thereafter but age at ART initiation varies from child to child. Time since diagnosis and duration on ART may be important factors associated with disclosure [33] and should be investigated in this setting. Another potential limitation of this study was the validity of the proxy- and self-reports for obtaining information on disclosure status, adherence, and experiences of stigma and depression. Validated measures for assessing disclosure status do not exist, so we attempted to use a variety of questionnaire items, evaluating potential aspects of disclosure such as whether the child knew their disease, the name of their disease, why they took medicines, or why they attended clinic. We used the PHQ-2 depression screening questions because they have reasonable validity and reliability among HIV-infected adults in western Kenya [34] and adolescents in the US [35] but there are no such studies on depression screening among children and adolescents in this setting. No validated measures for HIV-related stigma currently exist for this population. [36] Data related to disclosure were collected in the context of a routine HIV clinic visit, which limits the data points available. It is also possible that the caregivers or children may have been hesitant to discuss disclosure status, adherence, stigma, or depression symptoms with their regular clinician. Nonetheless, our intent was to make these discussions a routine part of the clinical encounter between clinician and family or patient, and asking these questions as part of the clinical visit modeled that patient-physician interaction. Finally, the cross-sectional design of this study did not allow us to measure disclosure rates of HIV status to children, causal pathways of disclosure or the potential impact of disclosure on clinical and psychosocial outcomes. Longitudinal cohort studies on disclosure of HIV status in this setting are urgently needed to answer these important questions as more HIV-infected children and adolescents make the difficult transition to adulthood.

## Conclusions

This sample from a large, pediatric HIV care program in sub-Saharan Africa suggests a low prevalence of disclosure of HIV status to children, while highlighting how disclosure may be related to key outcomes such as medication adherence, experiences of stigma, and symptoms of depression. More data are needed to better understand the impact of disclosure and to inform disclosure support interventions as children and their families go through this challenging process.
